# Enhancing astaxanthin accumulation in *Xanthophyllomyces dendrorhous* by a phytohormone: metabolomic and gene expression profiles

**DOI:** 10.1111/1751-7915.13567

**Published:** 2020-05-19

**Authors:** Xueshan Pan, Baobei Wang, Ran Duan, Jing Jia, Jun Li, Weide Xiong, Xueping Ling, Cuixue Chen, Xiaohong Huang, Guoliang Zhang, Yinghua Lu

**Affiliations:** ^1^ Department of Chemical and Biochemical Engineering College of Chemistry and Chemical Engineering Xiamen University Xiamen 361005 China; ^2^ College of Oceanology and Food Science Quanzhou Normal University Quanzhou 362000 China; ^3^ SDIC Biotechnology Investment Co. Ltd State Development and Investment Corporation Beijing 100034 China; ^4^ The Key Lab for Synthetic Biotechnology of Xiamen City Xiamen University Xiamen 361005 China; ^5^ Department of Stomatology Medical College Xiamen University Xiamen 361005 China; ^6^ Institute of Oceanic and Environmental Chemical Engineering State Key Lab Breeding Base of Green Chemical Synthesis Technology Zhejiang University of Technology Hangzhou 310014 China

## Abstract

*Xanthophyllomyces dendrorhous* is a promising source of natural astaxanthin due to its ability to accumulate high amounts of astaxanthin. This study showed that 6‐benzylaminopurine (6‐BAP) is an effective substrate that enhances cell biomass and astaxanthin accumulation in *X. dendrorhous*. In the current study, the biomass and astaxanthin content in *X. dendrorhous* were determined to be improved by 21.98% and 24.20%, respectively, induced by 6‐BAP treatments. To further understand the metabolic responses of *X. dendrorhous* to 6‐BAP, time‐course metabolomics and gene expression levels of *X. dendrorhous* cultures with and without 6‐BAP feeding were investigated. Metabolome analysis revealed that 6‐BAP facilitated glucose consumption, promoted the glycolysis, suppressed the TCA cycle, drove carbon flux of acetyl‐CoA into fatty acid and mevalonate biosynthesis, and finally facilitated the formation of astaxanthin. ROS analysis suggested that the antioxidant mechanism in *X. dendrorhous* can be induced by 6‐BAP. Additionally, the process of 6‐BAP significantly upregulated the expression of six key genes involved in pathways related to astaxanthin biosynthesis. This research demonstrates the metabolomic mechanism of phytohormone stimulation of astaxanthin production iNn *X. dendrorhous* and presents a new strategy to improve astaxanthin production to prevent the dilemma of choosing between accumulation of astaxanthin and cell biomass.

## Graphical abstract

6‐benzylaminopurine (6‐BAP) is an effective substrate that enhances cell biomass and astaxanthin accumulation in *X. dendrorhous*. This study provides a better understanding of the regulatory mechanism for enhancement of cell biomass and astaxanthin biosynthesis in *X. dendrorhous* by 6‐BAP and a new strategy to improve astaxanthin production by conquering the dilemma of choosing between accumulation of astaxanthin and cell biomass.

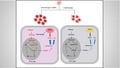

## Introduction

Astaxanthin is an important ketocarotenoid that is widely used in the food, aquaculture, nutraceutical and pharmaceutical industries because of its strong antioxidant activity (Rodríguez‐Sáiz *et al.*, 2010; Schmidt *et al.*, 2011) and numerous biological functions (Gong and Bassi, 2016). The global astaxanthin market has grown significantly in recent years (Gervasi *et al.*, 2019). The basidiomycetous red yeast *Xanthophyllomyces dendrorhous* (asexual state *Phaffia rhodozyma*) belongs to a basal lineage of the Agaricomycotina within the Tremellomycetes and possesses a set of unique characteristics of outstanding economic and ecological value (Golubev, 1995; Fell and Blatt, 1999; Bellora *et al.*, 2016; Gervasi *et al.*, 2018). This yeast is regarded as a potential strain to reduce the cost of natural astaxanthin due to its ability to accumulate large amounts of astaxanthin through high‐density fermentation and easy cultivation (Rodríguez‐Sáiz *et al.*, 2010, Bellora *et al.*, 2016).

Many studies have focused on increasing astaxanthin production through genetic engineering and optimization of fermentation conditions (Schmidt *et al.*, 2011; Gassel *et al.*, 2014; Hara *et al.*, 2014; Pan *et al.*, 2017). A yield of high astaxanthin content (5.1 mg g^−1^ DCW) by *X. dendrorhous* AXJ‐20 by lowering the pH during the bioprocess and increasing trace elements and vitamin concentrations has been reported (Schewe *et al.*, 2017). Wang et al. showed that glutamate feeding facilitated glucose consumption and further led to the increase in astaxanthin content in *X. dendrorhous* UV3‐721 (Wang *et al.*, 2019). Moreover, the literature shows that the cell growth and astaxanthin content of *X. dendrorhous* are mainly influenced by the carbon source, nitrogen concentration and Cu^2+^ (Martinez‐Moya *et al.*, 2015; Martinez‐Cardenas *et al.*, 2018). Recently, the addition of micronutrients (such as vitamins, trace elements and fungal elicitors) has been another crucial strategy to improve secondary metabolite production in microorganisms. It has been reported that butylated hydroxyanisole enhances astaxanthin content in *Haematococcus pluvialis* (Shang *et al.*, 2016) and that an elicitor prepared from *Rhodotorula glutinis* was able to increase astaxanthin yield by 90.60% in *X. dendrorhous* (Wang *et al.*, 2006).

Phytohormones (e.g. salicylic acid, ethylene and methyl jasmonate), a class of signalling molecules that act as stimulants, were demonstrated to stimulate a variety of biological processes, such as cell growth and the metabolism of fatty acids, carotenoids and carbohydrates in microalgae and fungi (Singh *et al.*, 2016; He *et al.*, 2017). The published literature shows that salicylic acid and 1‐aminocyclopropane‐1‐carboxylic acid (a precursor of ethylene) were effective on enhancing astaxanthin accumulation in *H. pluvialis* (Gao *et al.*, 2012) and that salicylic acid also facilitates carotenoid biosynthesis in *Tetraselmis suecica* (Ahmed *et al.*, 2015). In *H. pluvialis*, salicylic acid is supposedly a molecular signal in the network of carotenoid biosynthesis which leads to overexpression of carotenoid biosynthesis genes, such as *ipi*‐1, *psy*, *pds*, *crt*R‐B and *lyc*. Furthermore, 1‐aminocyclopropane‐1‐carboxylic acid further promotes cell growth and enhances astaxanthin accumulation on the basis of salicylic acid (Lee *et al.*, 2016). In addition, ethylene has been found to be effective in promoting β‐carotene biosynthesis in *Blakeslea trispora* by decreasing TCA flux and increasing the accumulation of acetyl‐CoA (He *et al.*, 2017).

It is well‐known that *X. dendrorhous* was isolated from trees in the mountain areas of Japan and Alaska (Phaff *et al.*, 1972). The yeast was proposed to have evolved as a result of its adaptation to metabolites of its symbiotic plants (Bellora *et al.*, 2016). However, the effects and mechanisms of phytohormones on cell biomass and astaxanthin content in *X. dendrorhous* have never been reported. A key synthetic cytokinin, 6‐benzylaminopurine (6‐BAP), not only participates in stimulating cell growth and fruit maturity by accelerating cell division (Carimi *et al.*, 2003) but also serves as an inhibitor of respiratory kinase in plants. It has been demonstrated that 6‐BAP‐treated spears had a better colour, firmness and overall appearance; moreover, they retained more chlorophyll and ascorbic acid and less fibre (An *et al.*, 2006). However, few studies of the effects of 6‐BAP on the growth of microorganisms have been reported (Lin *et al.*, 2017; Yu *et al.*, 2019). In this study, we found that 6‐BAP could promote astaxanthin production and increase the cell biomass of *X. dendrorhous*. Thus, 6‐BAP is probably a promising phytohormone which might help producers of astaxanthin to prevent the dilemma of choosing between astaxanthin accumulation and cell biomass.

Comparative metabolomics is one commonly used method to analyse intracellular metabolites and is regarded as a useful tool to provide new insights into understanding the regulatory mechanisms of microorganisms to environmental stimulus (Wang *et al.*, 2014; Alcalde and Fraser, 2018). In this study, gas chromatography‐mass spectrometry (GC‐MS) combined with quantitative real‐time PCR (RT‐qPCR) was applied to investigate the influence of 6‐BAP on cell biomass and astaxanthin accumulation and to explore its regulatory role in astaxanthin biosynthesis. The aim of this study was to provide a better understanding of the mechanism underlying the enhancement of both, cell biomass of *X. dendrorhous* and astaxanthin biosynthesis, by 6‐BAP.

## Results and discussion

### Effects of 6‐BAP on cell biomass and astaxanthin biosynthesis in *X. dendrorhous*

Our study showed that both cell biomass and astaxanthin biosynthesis in *X. dendrorhous* UV3‐721 were boosted by the addition of 0.25 mg l^−1^ 6‐BAP at 24 h after inoculation (Fig. S1). To further analyse the effects of 6‐BAP on cell biomass and to gain a better understanding of the metabolic responses of *X. dendrorhous* UV3‐721 to 6‐BAP, cell biomass characteristics, astaxanthin content, residual glucose and ammonium of the control group (culture without addition of 6‐BAP) and the 6‐BAP group (culture with addition of 0.25 mg l^−1^ 6‐BAP at 24 h) were compared.

**Fig. 1:**
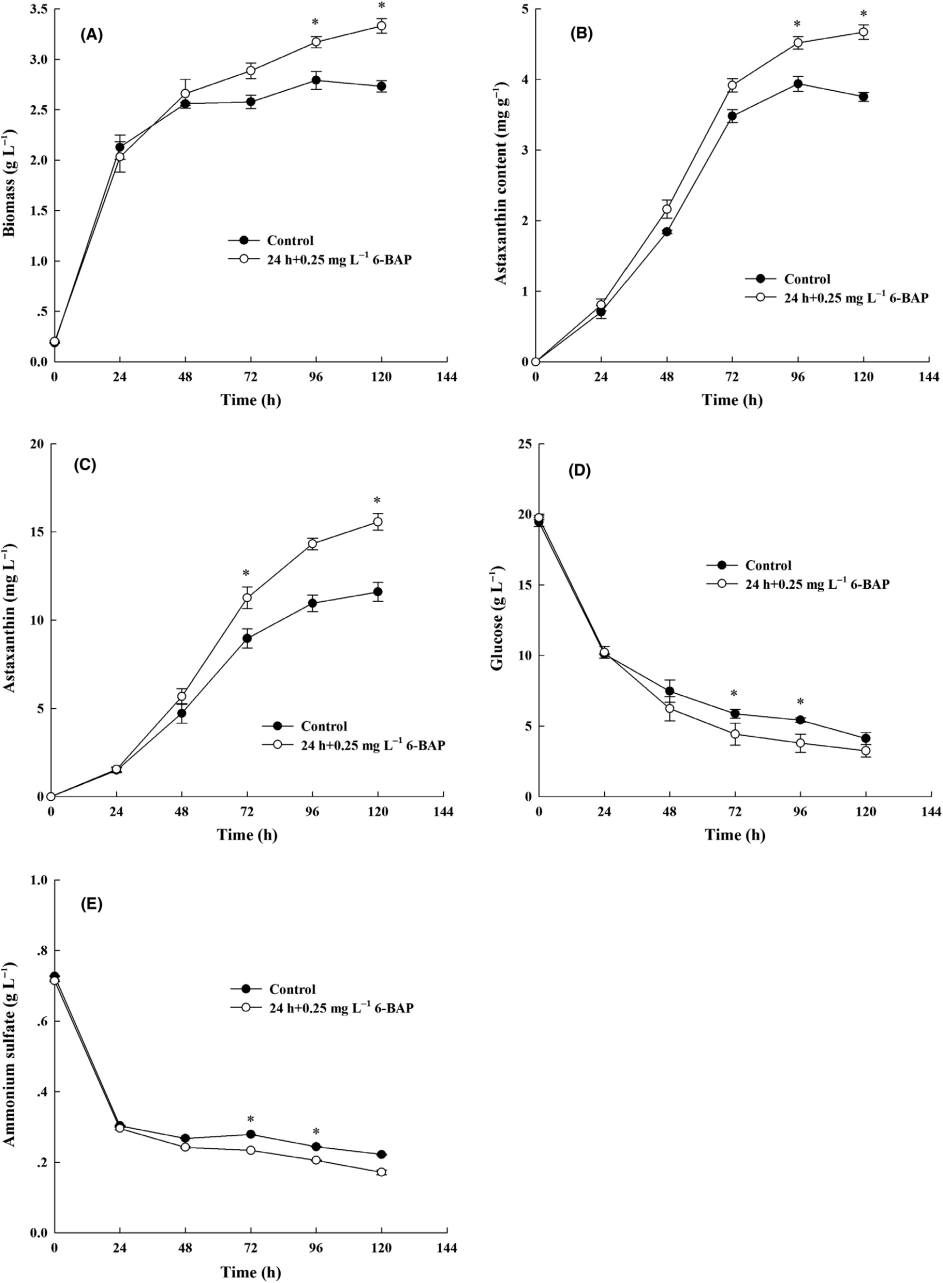
Time‐course profiles of *X. dendrorhous* UV3‐721 cultures in the 6‐BAP and control groups. Control group: solid circle symbols, 6‐BAP group: hollow circle symbols. (A) Biomass (g l^−1^); (B) astaxanthin content (mg g^−1^); (C) astaxanthin (mg l^−1^); (D) glucose (g l^−1^); (E) ammonium sulfate (g l^−1^), where * represents statistical differences with *P* < 0.05 compared with the control. Values are mean ± standard deviation of three independent experiments.

*X. dendrorhous* UV3‐721 cells of the 6‐BAP group continued to grow faster than those of the control group after the addition of 6‐BAP. When 0.25 mg l^−1^ 6‐BAP was added to the culture at 24 h of cultivation, biomass and astaxanthin content reached a maximum (3.33 g l^−1^ and 4.67 mg g^−1^ DCW) at 120 h. The values were 21.98% and 24.20% higher than those of the control group respectively (Fig. 1A,B). As a result, the astaxanthin yield of *X. dendrorhous* UV3‐721 increased by 34.11% at 120 h after 6‐BAP addition (Fig. 1C). It was worth noting that cell growth usually refers to cell proliferation, the increase in cell numbers that occurs through repeated cell division. In addition, cell growth can also refer to the enlargement of cell volume which can take place in the absence of cell division. The latter term seems to be the case in this study, since the increased biomass was correlated with an increased synthesis of fatty acids, sterols and trehalose among others (Table 1). These results indicated that an appropriate 6‐BAP feeding strategy might be a useful way to enhance astaxanthin production in *X. dendrorhous*. A widely known dilemma in *X. dendrorhous* is that its increase in astaxanthin content is usually accompanied by a reduction in biomass (Martinez‐Moya *et al.*, 2015). Importantly, 6‐BAP feeding seemingly allowed *X. dendrorhous* to avoid this dilemma.

**Table 1:** Metabolites responding to 6‐BAP treatment in *X. dendrorhous* UV3‐721.

Metabolites	48 h		72 h		96 h		120 h	
	Control	6‐BAP	Control	6‐BAP	Control	6‐BAP	Control	6‐BAP
Oxaloacetate	4.73 ± 0.35	3.18 ± 0.28*	5.12 ± 0.25	3.76 ± 0.28**	9.45 ± 2.08	8.03 ± 0.91	6.84 ± 0.50	5.48 ± 0.19**
Succinic acid	0.04 ± 0.02	0.02 ± 0.01	0.04 ± 0.02	0.02 ± 0.00**	0.40 ± 0.09	0.18 ± 0.05	0.38 ± 0.01	0.10 ± 0.02**
Fumaric acid	0.20 ± 0.04	0.19 ± 0.02	0.14 ± 0.04	0.13 ± 0.03**	0.07 ± 0.05	0.10 ± 0.06*	0.05 ± 0.01	0.08 ± 0.00
Citric acid	0.26 ± 0.01	0.39 ± 0.02*	0.14 ± 0.04	0.24 ± 0.03**	0.14 ± 0.02	0.25 ± 0.03	0.14 ± 0.02	0.26 ± 0.04
Malic acid	0.13 ± 0.01	0.17 ± 0.01	0.13 ± 0.03	0.14 ± 0.23	0.06 ± 0.04	0.09 ± 0.08*	0.08 ± 0.001	0.07 ± 0.08
d‐Glucose	408.73 ± 34.75	685.72 ± 26.35*	132.36 ± 19.69	351.67 ± 66.95**	182.55 ± 22.17	191.23 ± 12.46	179.17 ± 10.16	160.08 ± 6.23*
Lactic acid	2.84 ± 0.07	2.45 ± 0.33	3.14 ± 0.44	2.04 ± 0.41**	4.02 ± 1.12	4.99 ± 1.50	2.25 ± 0.06	2.44 ± 0.35
Phosphoric acid	0.81 ± 0.08	1.21 ± 0.31*	1.20 ± 0.02	1.47 ± 0.22*	1.30 ± 0.14	1.69 ± 0.31	1.64 ± 0.17	2.16 ± 0.14**
Ethanol	0.29 ± 0.03	0.19 ± 0.02	0.32 ± 0.07	0.27 ± 0.03	1.37 ± 0.05	0.81 ± 0.13*	0.83 ± 0.04	0.49 ± 0.03
Alanine	0.05 ± 0.01	0.09 ± 0.01**	0.08 ± 0.02	0.12 ± 0.02**	0.11 ± 0.06	0.14 ± 0.05	0.11 ± 0.00	0.15 ± 0.02**
Serine	0.11 ± 0.01	0.14 ± 0.03	0.15 ± 0.06	0.26 ± 0.04*	0.18 ± 0.01	0.21 ± 0.01**	0.14 ± 0.02	0.23 ± 0.03**
Threonine	0.05 ± 0.03	0.04 ± 0.00	0.09 ± 0.00	0.07 ± 0.01*	0.08 ± 0.05	0.09 ± 0.08	0.06 ± 0.01	0.07 ± 0.02
Tyramine	–	0.01 ± 0.00	0.24 ± 0.11	0.55 ± 0.10**	0.16 ± 0.07	0.10 ± 0.15	0.13 ± 0.01	0.09 ± 0.01**
Leucine	0.16 ± 0.02	0.5 ± 0.040**	1.91 ± 0.46	2.20 ± 0.29	0.20 ± 0.01	0.36 ± 0.04**	0.33 ± 0.12	0.35 ± 0.21
Glutamate	1.26 ± 0.07	1.04 ± 0.07**	1.45 ± 0.30	0.87 ± 0.10**	1.26 ± 0.82	1.09 ± 1.04	1.09 ± 0.06	1.15 ± 0.53
Aspartate	1.40 ± 0.18	1.64 ± 0.11	1.48 ± 0.76	1.47 ± 0.37	1.25 ± 0.27	0.83 ± 0.05**	2.09 ± 0.41	1.97 ± 0.58
Myristic acid	2.15 ± 0.06	2.50 ± 0.15**	1.98 ± 0.12	2.52 ± 0.43*	3.22 ± 0.25	3.69 ± 0.23*	3.28 ± 0.56	3.09 ± 0.35
Palmitic acid	8.64 ± 1.44	11.23 ± 1.07*	6.38 ± 1.68	8.54 ± 1.87*	8.86 ± 0.73	8.44 ± 0.92	7.92 ± 1.03	8.17 ± 0.88
9,12‐Octadecadienoic acid	6.27 ± 0.51	11.13 ± 1.32**	2.27 ± 1.09	1.67 ± 0.55*	4.71 ± 2.34	3.90 ± 1.04	3.21 ± 0.44	2.60 ± 0.47
9‐Octadecenoic acid	1.32 ± 0.15	1.69 ± 0.22*	1.50 ± 0.12	1.03 ± 0.35*	1.46 ± 0.20	1.23 ± 0.16	0.78 ± 0.10	1.17 ± 0.37
Stearic acid	3.80 ± 0.80	4.73 ± 0.57	5.80 ± 0.33	7.39 ± 1.11*	7.33 ± 0.75	7.48 ± 1.41	5.71 ± 0.38	7.81 ± 1.72*
Arachidic acid	0.06 ± 0.01	0.12 ± 0.01**	0.28 ± 0.07	0.27 ± 0.04	0.15 ± 0.09	0.17 ± 0.05	0.13 ± 0.05	0.30 ± 0.03**
Arabinose	1.07 ± 0.06	1.18 ± 0.09	0.63 ± 0.14	0.78 ± 0.12*	1.30 ± 0.14	1.29 ± 0.17	1.19 ± 0.08	1.19 ± 0.19
Xylose	0.38 ± 0.08	0.41 ± 0.05	0.69 ± 0.20	0.62 ± 0.32	0.64 ± 0.14	0.59 ± 0.20*	0.87 ± 0.13	0.83 ± 0.15
3‐α‐Mannobiose	18.99 ± 1.81	13.84 ± 1.64**	8.58 ± 1.60	11.73 ± 1.81*	15.83 ± 1.28	11.64 ± 1.20**	17.48 ± 2.61	11.67 ± 0.92*
Myo‐inositol	3.51 ± 0.58	6.77 ± 0.74**	2.25 ± 0.46	8.38 ± 1.21**	2.39 ± 0.57	3.57 ± 2.52	3.29 ± 0.72	7.10 ± 0.45
Trehalose	50.82 ± 3.81	61.7 ± 6.89*	57.83 ± 9.05	79.39 ± 9.78**	74.09 ± 17.18	84.17 ± 9.81	79.76 ± 6.26	81.24 ± 14.95
Cellobiose	0.10 ± 0.02	0.21 ± 0.08*	1.16 ± 1.66	1.27 ± 1.59	0.79 ± 0.54	0.58 ± 0.28	0.43 ± 0.06	1.14 ± 0.55*
Uracil	0.34 ± 0.02	0.32 ± 0.04	0.33 ± 0.08	0.28 ± 0.06	1.24 ± 0.38	0.91 ± 0.67*	1.01 ± 0.06	0.75 ± 0.24
5‐Methylcytosine	0.18 ± 0.01	0.25 ± 0.02**	0.10 ± 0.04	0.13 ± 0.03	0.29 ± 0.04	0.26 ± 0.03	0.22 ± 0.01	0.23 ± 0.02
3‐Hydroxybutyric acid	0.05 ± 0.02	0.10 ± 0.02**	0.03 ± 0.09	0.06 ± 0.02*	0.11 ± 0.06	0.17 ± 0.15	0.21 ± 0.02	0.22 ± 0.01
Propanoic acid	0.13 ± 0.02	0.12 ± 0.02	0.16 ± 0.05	0.14 ± 0.05	0.66 ± 0.04	0.56 ± 0.03**	0.53 ± 0.03	0.41 ± 0.07*
Urea	1.06 ± 0.17	0.99 ± 0.02	0.78 ± 0.42	0.87 ± 0.42	1.72 ± 0.58	2.88 ± 2.51*	0.72 ± 0.16	1.22 ± 0.67
Phosphorylethanolamine	1.41 ± 0.18	1.58 ± 0.20	1.55 ± 0.37	2.50 ± 0.21**	1.30 ± 0.39	1.23 ± 0.38	2.28 ± 0.43	1.54 ± 0.07**
Farnesol	0.03 ± 0.01	0.05 ± 0.00**	0.05 ± 0.01	0.08 ± 0.01**	0.16 ± 0.15	0.18 ± 0.11	0.04 ± 0.01	0.05 ± 0.01
Squalene	0.03 ± 0.01	0.05 ± 0.01*	0.08 ± 0.01	0.12 ± 0.03*	0.13 ± 0.07	0.16 ± 0.03	0.08 ± 0.01	0.14 ± 0.05
Ergosterol	4.04 ± 0.34	5.15 ± 0.78	3.47 ± 0.61	4.93 ± 0.74*	4.67 ± 0.06	4.96 ± 0.91	4.13 ± 0.11	4.33 ± 0.83
Ergosta‐7,22‐dien‐3‐ol	0.35 ± 0.14	0.48 ± 0.08*	0.34 ± 0.07	0.43 ± 0.07	0.61 ± 0.27	0.63 ± 0.18	0.26 ± 0.05	0.57 ± 0.18*
Lanosterol	0.05 ± 0.03	0.09 ± 0.02	0.05 ± 0.01	0.08 ± 0.01*	0.09 ± 0.10	0.09 ± 0.04	0.06 ± 0.01	0.12 ± 0.03*

The data represent the metabolite content (mg g^−1^ DCW) and are shown as the averages of 6 replicates ± SD. Statistical significance was estimated by t‐test.

* *P* < 0.05 compared with the control; **P < 0.01 compared with the control.

In addition, glucose and ammonium sulfate were consumed quickly during the first 24 h after inoculation in both cultures (more precisely, the consumption rate of glucose and ammonium sulfate was 0.39 g l^−1^ h^−1^ and 0.02 g l^−1^ h^−1^ respectively) (Fig. 1D,E). Consumption rate of glucose was tested every 24 hours after adding 6‐BAP for 24 h, and the consumption rate of glucose in both cultures was slowed. However, the maximal consumption rate of glucose was 0.17 g l^−1^ h^−1^ in the 6‐BAP group, which was 54.55% higher than that of the control group (0.11 g l^−1^ h^−1^). Similarly, the consumption rate of ammonium sulfate in both cultures slowed after 24 h, but cells fed with 6‐BAP had a faster ammonium sulfate consumption rate (2.23 mg l^−1^ h^−1^) than cells in the control group (1.49 mg l^−1^ h^−1^). Residual glucose and ammonium sulfate concentration at 120 h was 3.25 g l^−1^ and 0.17 g l^−1^ for the 6‐BAP treatment group and was 4.12 and 2.22 g l^−1^ for the control group respectively (Fig. 1D,E). These results indicated that 6‐BAP stimulated *X. dendrorhous* to assimilate carbon and nitrogen from the medium. Based on this phenomenon, metabolome analysis was carried out to find changes caused by 6‐BAP at the metabolite level in *X. dendrorhous*.

### Effect of 6‐BAP on the metabolome of *X. dendrorhous*

**Fig. 2:**
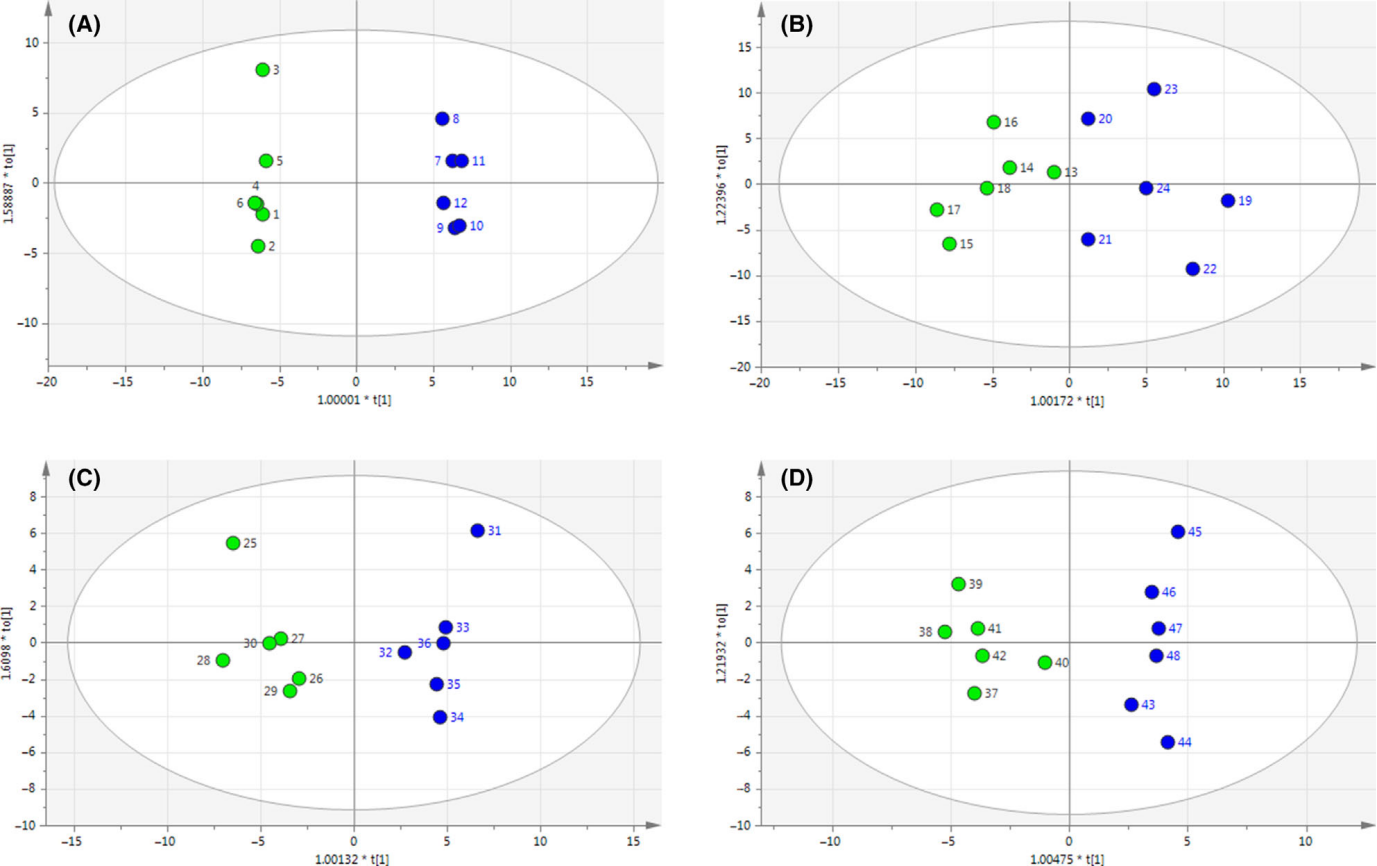
PLS‐DA derived plots for pairwise comparisons between the 6‐BAP and control groups at various time points: (A) 48 h, (B) 72 h, (C) 96 h and (D) 120 h. Green circle: control groups. Blue circle: 6‐BAP treatment groups.

**Fig. 3:**
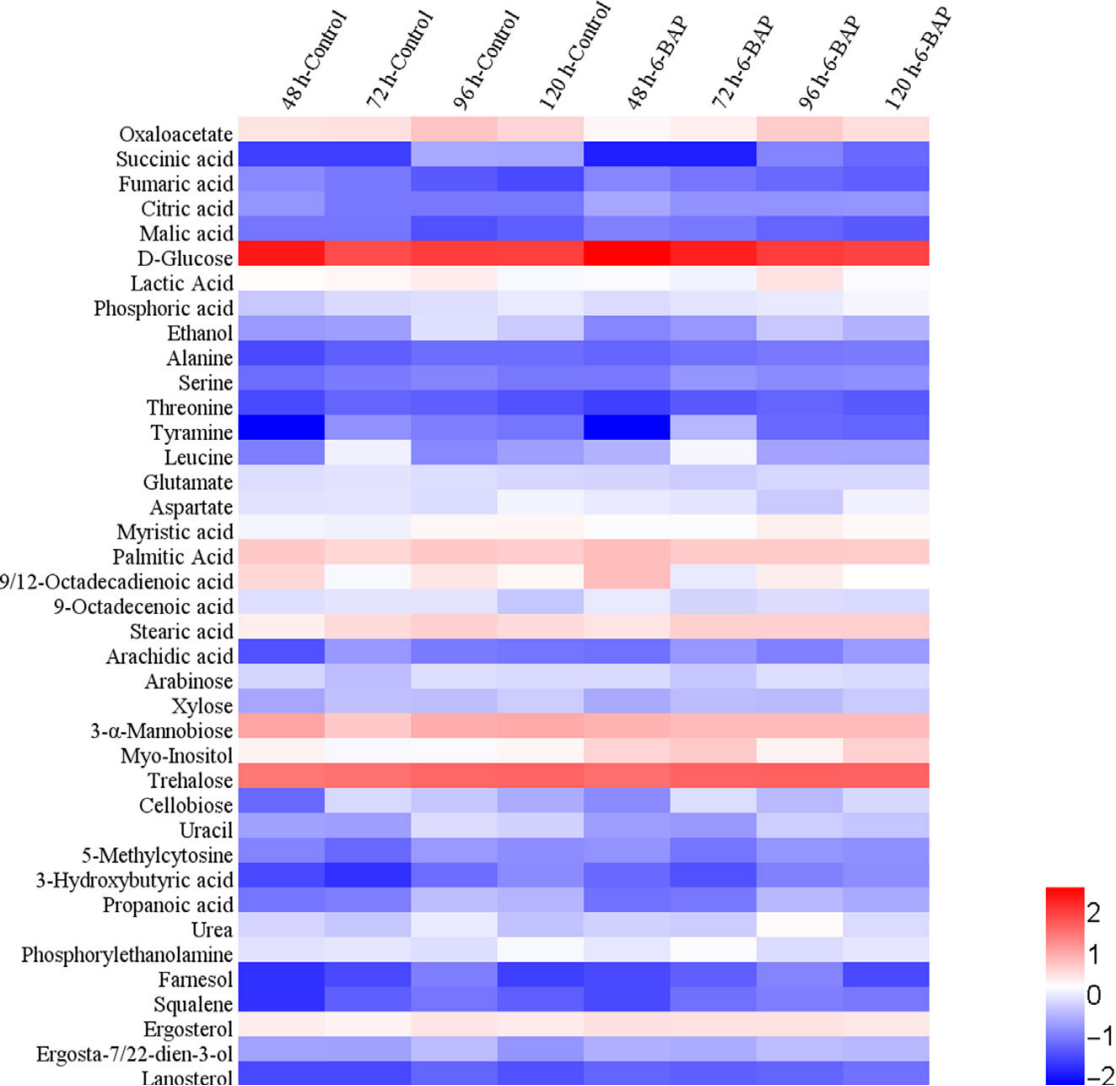
Hierarchical cluster analysis (HCA) for identified metabolites. All data are expressed as the means of six replicates. For each metabolite, the response ratio was normalized to log10.

Metabolites of the 6‐BAP group and the control group of *X. dendrorhous* UV3‐721 cells were analysed by GC‐MS. The results revealed that metabolomic profiles of the control group and the 6‐BAP group were well‐separated at all four time points (Fig. 2). The PLS‐DA analysis was conducted with 6 replicates from separate yeast cultures. As shown in Fig. 3, Tables 1 and 2, a total of 39 chemically classified metabolites with a very important variable of projection (VIP) value >1 and *P* values <0.05 were analysed. Most of them were involved in the tricarboxylic acid (TCA) cycle, carbohydrate metabolism, fatty acid synthesis and amino acid metabolism. Functional classification is shown in the heat map of Fig. 3.

**Table 2:** Relative changes in the cell metabolite abundance when *X. dendrorhous* was cultured with 6‐BAP versus control.

Metabolites	Fold change 6‐BAP/Control^a^				Metabolites	Fold change 6‐BAP/Control^a^			
	48 h	72 h	96 h	120 h		48 h	72 h	96 h	120 h
*TCA cycle*									
Oxaloacetate	0.672	**0.734**	0.850	**0.801**	Stearic acid	1.245	1.274	1.020	1.368
Succinic acid	0.500	**0.500**	0.450	**0.263**	Arachidic acid	**2.000**	0.964	1.133	**2.308**
Fumaric acid	0.950	**0.929**	1.429	1.600	*Carbohydrate metabolism*				
Citric acid	1.500	**1.714**	1.786	1.857	Arabinose	1.103	1.238	0.992	1.000
Malic acid	1.308	1.077	1.500	0.875	Xylose	1.079	0.899	0.922	0.954
*Glycolysis pathway*					3‐α‐Mannobiose	**0.729**	1.368	**0.735**	**0.668**
d‐Glucose	1.678	**2.657**	1.048	0.893	Myo‐inositol	**1.929**	**3.724**	1.494	2.158
Lactic acid	0.863	**0.650**	1.241	1.084	Trehalose	1.214	**1.373**	1.136	1.019
Phosphoric acid	1.494	1.225	1.300	**1.317**	Cellobiose	2.100	1.095	0.734	2.651
Ethanol	0.655	0.844	0.591	0.590	*Nucleotide*				
*Amino acids*					Uracil	0.941	0.848	0.734	0.743
Alanine	**1.800**	**1.500**	1.273	**1.364**	5‐Methylcytosine	**1.389**	1.300	0.897	1.045
Serine	1.273	1.733	**1.167**	**1.643**	*Organic acids*				
Threonine	0.800	0.778	1.125	1.167	3‐Hydroxybutyric acid	**2.000**	2.000	1.545	1.048
Tyramine	–	**2.292**	0.625	**0.692**	Propanoic acid	0.923	0.875	**0.848**	0.774
Leucine	**3.125**	1.152	**1.800**	1.061	*Others*				
Glutamate	**0.825**	**0.600**	0.865	1.055	Urea	0.934	1.115	1.674	1.694
Aspartate	1.171	0.993	**0.664**	0.943	Phosphorylethanolamine	1.121	**1.613**	0.946	**0.675**
*Fatty acids*					Farnesol	**1.697**	**1.590**	1.109	1.093
Myristic acid	**1.163**	1.273	1.146	0.942	Squalene	1.667	1.500	1.231	1.750
Palmitic acid	1.300	1.339	0.953	1.032	Ergosterol	1.275	1.421	1.062	1.048
9,12‐Octadecadienoic acid	**1.775**	0.736	0.828	0.810	Ergosta‐7,22‐dien‐3‐ol	1.371	1.265	1.033	2.192
9‐Octadecenoic acid	1.280	0.687	0.842	1.500	Lanosterol	1.800	1.600	1.000	2.000

^a^ Mean fold changes in the 6‐BAP group compared with the control group. Data in the table were shown as the averages of six replicates. Statistical significance was estimated by t‐test (*P* < 0.01) which is shown as bold value.

### 6‐BAP fluctuates glycolysis pathway in *X. dendrorhous*

As shown in Tables 1 and 2, the content of intracellular glucose in cells with 6‐BAP was increased by 67.8% (*P* < 0.05) and 167.69% (*P* < 0.01) at 48 and 72 h respectively. The increase in intracellular glucose in the first 72 h is consistent with the result that 6‐BAP promoted cells to assimilate glucose from the medium (Fig. 1D).

The temporary increase in intracellular glucose in cells was probably because the intracellular consumption rate of glucose in cells was slower than the glucose uptake rate by cells. The intracellular glucose content (mg g^−1^ DCW) in the 6‐BAP group returned to the same level as that in the control group from 96 h. From 48 to 120 h, the change in the intracellular glucose content (the value at 48 h minus the value at 120 h) in the 6‐BAP was 2.29‐fold higher than that of the control group (Table 1), indicating the use of the intracellular glucose was strengthened by 6‐BAP. Therefore, the glycolysis flux in cells was stimulated to make use of glucose to synthesize other metabolites.

Through the glycolysis pathway, one molecule of glucose breaks into two molecules of pyruvate. Thus, an increase in intracellular glucose might lead to the accumulation of pyruvate but no significant changes of pyruvate were found between the control group and the 6‐BAP group. However, the content of three amino acids generated from pyruvate (alanine, serine and leucine) was all significantly higher in the 6‐BAP group than in the control group in the first 48 h. In addition, lower levels of lactic acid and ethanol, two fermentation products derived from pyruvate, were found in the 6‐BAP group in the first 72 h. Compared with the control group, the content of lactic acid and ethanol in the 6‐BAP group decreased by 35.0% (*P* < 0.01) and 16.6% at 72 h respectively. These results indicated that more pyruvate was utilized to synthesize amino acids and less pyruvate flowed into the formation of lactic acid and ethanol. As Yu et al. reported, 6‐BAP could enhance the accumulation of DHA in *Aurantiochytrium* sp.YLH70, this study showed that pyruvate was the precursor for many metabolites, such as valine and ethanol, which were decreased in response to 6‐BAP, and more metabolic flux was directed to acetyl‐CoA (Yu *et al.*, 2016).

### 6‐BAP suppresses the TCA cycle in *X. dendrorhous*

The content of three important intermediates of the TCA cycle (oxaloacetate, succinic acid and fumaric acid) in the 6‐BAP group was lower than that in the control group during the entire course of cultivation (Tables 1 and 2). The content of oxaloacetate and fumaric acid reached the lowest level (26.6% and 7.1% decrease) at 48 h after treatment with 6‐BAP, while the content of succinic acid had its largest decrease after treatment by 6‐BAP for 120 h (73.7% decrease; Table 2). While the amount of citric acid was higher during the whole growth stage, malic acid content remained higher in the first 96 h in the 6‐BAP groups than in the control group.

The decrease in these crucial intermediates of the TCA cycle and the accumulation of citric acid indicated that the TCA cycle was inhibited by 6‐BAP. As it is well known, in the oleaginous yeasts, low TCA activity may promote citrate accumulation in the mitochondria and its subsequent transport to the cytosol. Citric acid is the first product in the TCA cycle, which is synthesized from acetyl‐CoA and oxaloacetate. Conversely, in the cytoplasm, citric acid can also produce acetyl‐CoA and oxaloacetate under the catalysis of the key cytoplasmic enzyme ATP citrate lyase (ACL). Therefore, citrate is considered to be the precursor of acetyl‐CoA for fatty acid synthesis in oleaginous yeasts and fungi (Evans *et al.*, 1983; Ratledge and Wynn, 2002), and citrate is a probable precursor of astaxanthin synthesis (Chavez‐Cabrera *et al.*, 2010). Evans et al. have reported that the supply of a moderate amount of citrate to the cytosol is an important control in the accumulation of lipids by yeasts (Evans *et al.*, 1983). Thus, data suggested that high citric acid concentration would be a sign of its excess for subsequent steps in the TCA cycle and its subsequent transport to the cytosol for acetyl‐CoA and oxaloacetate production. In addition, the content of four amino acids, namely threonine, tyramine, glutamate and aspartate, which are derived from intermediates of the TCA cycle, decreased in the 6‐BAP group at different time points (Table 1). This finding further suggested that the TCA cycle was inhibited.

Acetyl‐CoA is a key substrate for diverse cellular processes such as fatty acid biosynthesis, sterol biosynthesis and carotenoid biosynthesis (Venkateshwaran *et al.*, 2015). In *X. dendrorhous*, carotenoid biosynthesis begins with the formation of isopentenyl pyrophosphate (IPP) from acetyl‐CoA through the mevalonate pathway. The accumulation of large amounts of citric acid due to the addition of 6‐BAP will inhibit the TCA cycle to some extent. Subsequently, suppression of the TCA cycle made more acetyl‐CoA available for other metabolic pathways, such as fatty acid biosynthesis and the mevalonate pathway. Consistently, Li et al. also mentioned that a decrease in the TCA cycle can drive central carbon flux into fatty acid and mevalonate biosynthesis (Li *et al.*, 2018).

### 6‐BAP promotes fatty acid biosynthesis and the mevalonate pathway in *X. dendrorhous*

Fatty acids are the main components of the cell membrane and contribute greatly to their fluidity and permeability (Los *et al.*, 2013). Fatty acids also serve as a carbon storage source for cells. Considering their importance, predominant fatty acids, such as myristic acid, palmitic acid, 9, 12‐octadecadienoic acid, 9‐octadecenoic acid, stearic acid and arachidic acid, were qualified and quantified. As shown in Table 1, the total content of these fatty acids at 48 h in the 6‐BAP group was 31.4 mg g^−1^, 41.2% higher than that of the control group. Among all the detected fatty acids in *X. dendrorhous* UV3‐721, palmitic acid and 9, 12‐octadecadienoic acid underwent the biggest change, increasing by 29.98% and 77.51% respectively. These results suggested that 6‐BAP facilitated the accumulation of fatty acids in *X. dendrorhous* UV3‐721. The increase in fatty acids, especially unsaturated fatty acids, would enhance the fluidity and permeability of cell membranes and assist cells in adapting to phytohormone (6‐BAP) conditions. These findings are quite similar to the results of Yu et al., who reported that 6‐BAP could enhance fatty acid production in *Aurantiochytrium* sp.YLH70 (Yu *et al.*, 2016).

Interestingly, the content of ergosterol in cells with 6‐BAP treatment was 1.3‐fold and 1.4‐fold of that in the control group at 48 and 72 h respectively. In addition, squalene and ergosta‐7, 22‐dien‐3‐ol, two intermediate products in the ergosterol biosynthesis pathway, also increased by 20% to 70% when treated with 6‐BAP within the first 72 h (Table 2). These findings suggested that biosynthesis of ergosterol in *X. dendrorhous* UV3‐721 was also stimulated by 6‐BAP treatment, which might indirectly reflect enhancement of the mevalonate pathway.

**Fig. 4:**
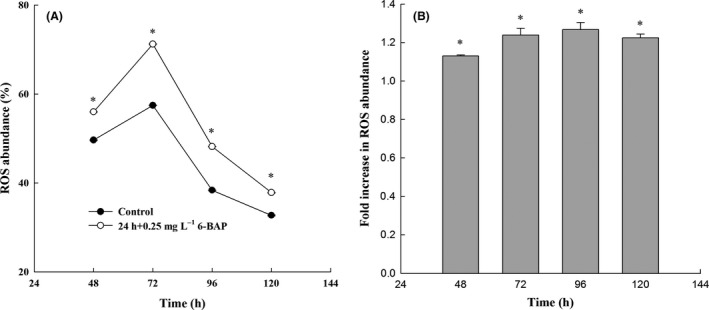
Intracellular reactive oxygen species (ROS) abundance. ss (A) Variation in ROS abundance with time of incubation; (B) fold increase in ROS abundance, where * represents statistical differences with *P* < 0.05 compared with the control. The solid circle and hollow circle represent the intracellular ROS abundance in the control group and the 6‐BAP group respectively. Values are mean ± standard deviation of three independent experiments.

It was worth noting that cellular fatty acid content in the 6‐BAP group started to decrease at 72 h when glucose was limited, while biomass, sterol and astaxanthin content kept increasing (Table 1). At the same time, ROS abundance reached a maximum (Fig. 4A). This finding probably occurred because during the later course of cultivation, acetyl‐CoA, a common precursor of fatty acids and astaxanthin, was mainly used to synthesize astaxanthin to resist high oxidative stress, leading to the decrease in fatty acids.

### 6‐BAP enhances oxidative stress in *X. dendrorhous*

Astaxanthin is a scavenger of free radicals, a chain‐breaking antioxidant and potent quencher of reactive oxygen species (ROS), such as singlet oxygen (^1^O_2_), superoxide ion (O_2_^−.^) and hydrogen peroxide (H_2_O_2_), irrespective of whether these are found in the yeast natural habitat or generated by the yeast itself through its intracellular oxidative metabolism (Alesci *et al.*, 2015; Martinez‐Cardenas *et al.*, 2018). The presence of this carotenoid is equivalent to higher survival capacity of the cells since it enhances resistance of the cell to oxidative stress (Cuellar‐Bermudez *et al.*, 2015). Thus, the biosynthesis of carotenoids serves as a survival strategy under oxidative stress for *X. dendrorhous* (Gessler *et al.*, 2007). Over the entire cultivation, significant increases in myo‐inositol and trehalose, two carbohydrate metabolism intermediates, were observed (Tables 1 and 2). Myo‐inositol is a growth factor for yeast and plays an important role in responding to environmental stresses, such as oxygen and osmotic pressure in *Aurantiochytrium* sp. and *Schizochytrium* sp. (Jakobsen *et al.*, 2007; Yu *et al.*, 2016). Interestingly, in the present study, the addition of 6‐BAP caused a significant increase in myo‐inositol (1.5‐ to 3.7‐fold), which might be a key stress indicator in response to 6‐BAP treatment. Moreover, trehalose, another stress indicator, significantly increased in the present study. Accumulation of trehalose is considered a defence mechanism against a variety of stress conditions including the presence of ROS (Weeks *et al.*, 2006). These findings were consistent with previous studies showing that *X. dendrorhous* with high content of trehalose exhibited a strong antioxidant response and accumulated large amounts of astaxanthin (Martinez‐Moya *et al.*, 2015). Therefore, myo‐inositol and trehalose were increased by 6‐BAP addition. Further investigation will be necessary to determine the relationship between 6‐BAP addition and myo‐inositol/trehalose metabolism.

A ROS detection assay was performed to establish the involvement of ROS abundance on astaxanthin. As we can see from Fig. 4A, following treatment with 6‐BAP, a significant difference in the level of ROS was detected after a 24‐h treatment. The ROS level was higher from 48 to 120 h in the 6‐BAP group than in the control group. At 48 h, the ROS content in the control group and the 6‐BAP group was 49.6% and 56.0% respectively. After 72 h of growth, the ROS content in the 6‐BAP treatment group was 71.2%, and only 57.4% in the control group. With the production of astaxanthin, which can scavenge ROS, the level of intracellular ROS is gradually reduced. However, at 96 h, the ROS was 1.27‐fold higher than that of the control group (Fig. 4B). These results suggest that 6‐BAP induces ROS accumulation, increases redox signalling and further induces synthesis of astaxanthin in *X. dendrorhous*.

### Transcriptional responses of genes involved in astaxanthin biosynthesis

**Fig. 5:**
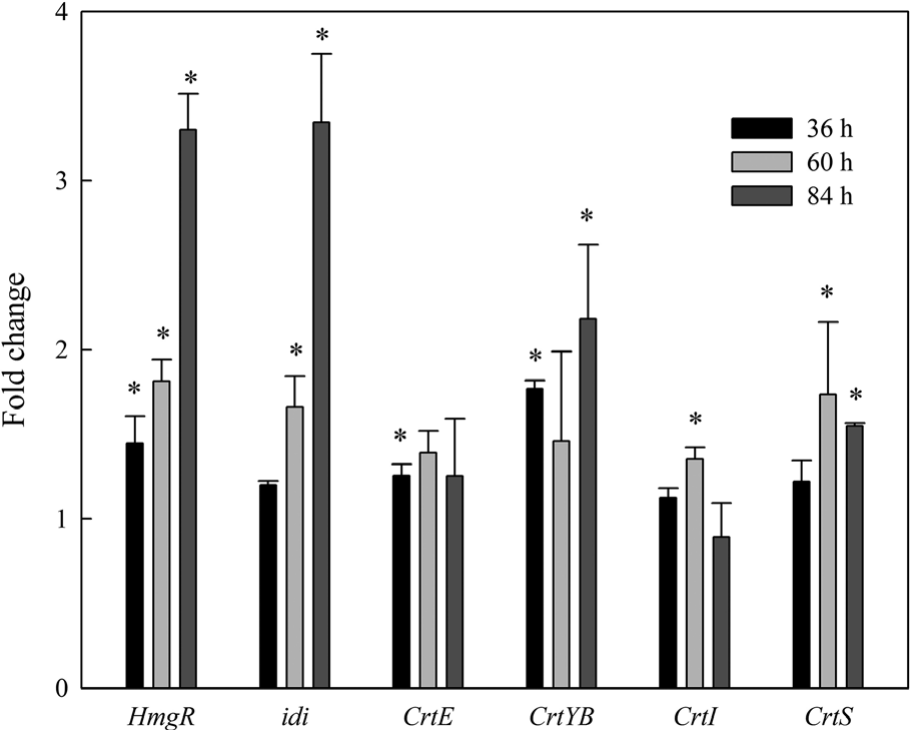
6‐BAP regulates the transcriptional level of key genes involved in astaxanthin synthesis. The key genes include *hmgR* (encoding HMG‐CoA reductase), *idi* (encoding IPP isomerase), *crtE* (encoding GGPP synthase), *crtYB* (encoding phytoene synthase/lycopene cyclase), *crtI* (encoding phytoene dehydrogenase) and *crtS* (encoding astaxanthin synthase), where * represents statistical differences with *P* < 0.05 compared with the control. Values are mean ± standard deviation of three independent experiments.

To explore the molecular mechanisms underlying the higher level of astaxanthin accumulation induced by 6‐BAP, RT‐qPCR was used to measure the expression of genes involved in the astaxanthin biosynthesis pathway. Genes encoding six key enzymes involved in carotenoid biosynthesis were analysed. HMG‐CoA reductase (*hmg*R), a rate‐limiting enzyme of the mevalonate pathway, catalyses HMG‐CoA to mevalonate (Hara *et al.*, 2014). As shown in Fig. 5, transcription of *hmgR* significantly increased with 6‐BAP treatment during the whole cultivation period. IPP isomerase (*idi*) catalyses the conversion of the relatively non‐reactive IPP to the highly reactive electrophile dimethylallyl pyrophosphate (DMAPP) (Kajiwara *et al.*, 1997). Transcription of *idi* was promoted by 6‐BAP at 60 h and 84 h, which might lead to the increase in the terpenoid precursor DMAPP. *crtE* encodes GGPP synthase, and *crtYB* encodes a bifunctional enzyme (phytoene synthase or lycopene cyclase). The transcription level of *crtE* was elevated by 6‐BAP at 36 h. Compared with the control group, transcription of *crtYB* in the 6‐BAP group was increased at 36 h (1.77‐fold) and 84 h (2.18‐fold) respectively. In the 6‐BAP groups, the expression of *crtI* was increased at 60 h (1.35‐fold), but it subsequently decreased to a basal level. The transcript level of *crtS,* one gene encoding key enzymes involved in the biosynthesis of astaxanthin, was higher at 60 h (1.74‐fold) and 84 h (1.55‐fold) in the 6‐BAP group than that in the control group. Gassel et al. suggested that genetic engineering of the complete pathway to improve the supply of all precursors for carotenogenesis can enhance the accumulation of astaxanthin (Gassel *et al.*, 2014). In this study, as a response to 6‐BAP, the transcription of genes in the mevalonate pathway and carotenoid biosynthesis was upregulated, which is consistent with the enhancement of astaxanthin accumulation in *X. dendrorhous* UV3‐721.

Interestingly, though the transcript levels of *hmgR*, *idi* and *crtYB* increased more than twofold in the 6‐BAP group over those in the control group, the transcript levels of *crtI* and *crtS* did not show such a strong increase. This observation indicated that astaxanthin biosynthesis was not singly regulated by gene overexpression; it was probably regulated using a hybrid strategy. For example, for the upstream pathway, enzymes (*hmgR*, *idi* and *crtYB*) are regulated at the transcript level, while for the downstream pathway, enzymes (*crtI* and *crtS*) are not only regulated at the transcript level, but might also be regulated at the protein level (enzyme activity).

### Possible regulatory mechanism through which 6‐BAP promotes the accumulation of astaxanthin in *X. dendrorhous*

**Fig. 6:**
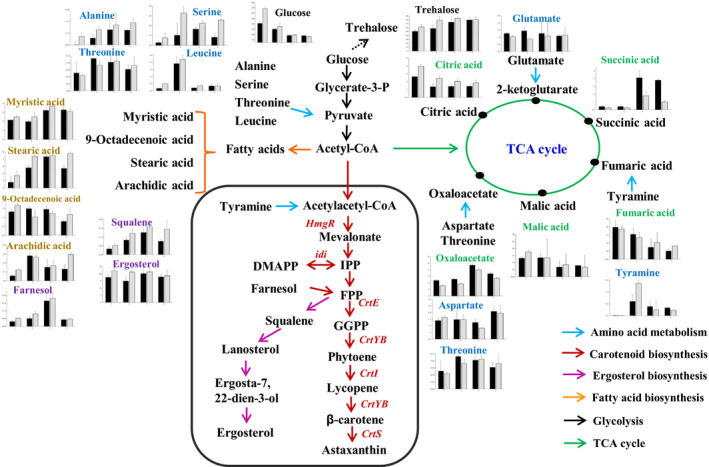
Metabolites change induced by 6‐BAP in *X. dendrorhous* UV3‐721. Change in the levels of intermediates was detected by GC‐MS and calculated by normalization of the peak area of each metabolite to the internal standard. The *x*‐axis in the graphs represents time (h), and the *y*‐axis represents concentration (mg g^−1^ DCW). The black and grey bars represent metabolites in the control and 6‐BAP treatment groups respectively. The red font indicates the key genes of carotenoid biosynthesis.

The comparison of the metabolites of the 6‐BAP group and the control group showed that metabolites content involved in the glycolysis pathway, TCA cycle and lipid biosynthesis were changed substantially in response to 6‐BAP in *X. dendrorhous* UV3‐721. The induced changes in metabolites by 6‐BAP in *X. dendrorhous* UV3‐721 were shown in Fig. 6. It was mapped out to characterize *X. dendrorhous* intracellular metabolism in response to 6‐BAP based on previous research (Martinez‐Moya *et al.*, 2015; Wang *et al.*, 2019). Glucose could pass through cell membranes and be used for cell metabolism. 6‐BAP induced cells to assimilate glucose from the medium. From 24 to 96 h, the consumption rate of intracellular glucose in the 6‐BAP group was higher than that of the control group, indicating that the glycolysis flux was induced by 6‐BAP. More importantly, astaxanthin is synthesized in *X. dendrorhous* via the mevalonate pathway, in which HMG‐CoA reductase (*hmgR*) is a rate‐limiting enzyme. HMG‐CoA reductase catalyses the transformation of HMG‐CoA to mevalonate. The literature shows that enhanced provision of precursors for carotenogenesis via the mevalonate pathway was attempted by overexpressing *hmgR* (Gassel *et al.*, 2014). In the current study, as a response to 6‐BAP, the significant increase in *hmgR* transcription suggested that the mevalonate pathway is increased, which is consistent with the enhancement of astaxanthin accumulation in *X. dendrorhous* UV3‐721. Additionally, the supply of 6‐BAP significantly upregulated the expression of six key genes involved in carotenogenesis biosynthesis. At the same time, 6‐BAP by impeding the TCA cycle or inhibiting the electron flow through the mitochondrial respiratory chain promotes citrate build‐up in mitochondria and its export into the cytosol. While the accumulation of citrate could inhibit the utilization of acetyl‐CoA by the TCA cycle resulting in the increased cellular acetyl‐CoA concentration. On the other hand, the activity of ATP citrate lyase (a cytosolic enzyme) may also contribute to the increased cellular acetyl‐CoA concentration via breaking down accumulated citrate. The synthesis of fatty acids, carotenoids and sterols apparently takes place in the cytosol (at least the former stages), using as common precursors including acetyl‐CoA, NADPH and ATP. This may imply that acetyl‐CoA availability in the cytosol jointly promotes the synthesis of all these compounds. For instance, the increased glycolytic flux might suggest that more glucose goes through the pentose phosphate pathway to supply the increased demand of NADPH required for the lipid synthesis and the increased ROS. As a consequence, more carbon sources (e.g. acetyl‐CoA) are available for astaxanthin or fatty acid biosynthesis in *X. dendrorhous*. Furthermore, intracellular ROS analysis suggested that 6‐BAP induces ROS accumulation, which increases redox signalling and further induces synthesis of astaxanthin in *X. dendrorhous*.

Overall, the mechanism proposed that with the treatment of 6‐BAP, the use of glucose for fermentation was increased, the glycolytic flux was promoted, and metabolites in fatty acids biosynthesis and mevalonate pathway were increased, while most metabolites in TCA cycle were decreased in *X. dendrorhous*. As a consequence, more carbon sources (e.g. acetyl‐CoA) are available for astaxanthin biosynthesis. Upregulation of several genes encoding key enzymes (e.g. IPP isomerase, phytoene synthase) involved in carotenogenesis biosynthesis might also be caused by the increase in their substrates and higher levels of ROS due to the supply of 6‐BAP. When the carbon source in the medium was exhausted, cellular fatty acid content in the 6‐BAP group started to decrease at 72 h, this finding probably occurred because during the later course of cultivation, acetyl‐CoA, a common precursor of fatty acids and astaxanthin, was mainly used to synthesize astaxanthin to assist cells in reducing oxidative damage. The continuous upregulation of several enzymes related to astaxanthin biosynthesis (*hmgR* and *idi*) further supports this assumption.

## Conclusions

This study demonstrated that 6‐benzylaminopurine (6‐BAP) could enhance cell biomass and astaxanthin accumulation in *X. dendrorhous*. The increased biomass was correlated with an increased synthesis of fatty acids, sterols and trehalose among others. Metabolome analysis revealed 6‐BAP facilitated intracellular glucose consumption, promoted the metabolic flux of glycolysis and inhibited TCA cycle, which further drove carbon flux (e.g. acetyl‐CoA) into fatty acid and mevalonate biosynthesis to improve the formation of astaxanthin. Moreover, the induced high ROS level by 6‐BAP could stimulate astaxanthin biosynthesis to increase the antioxidant function in *X. dendrorhous* to scavenge ROS. This study provides a better understanding of the regulatory mechanism for enhancement of cell biomass and astaxanthin biosynthesis in *X. dendrorhous* by 6‐BAP and a new strategy to improve astaxanthin production by conquering the dilemma of choosing between accumulation of astaxanthin and cell biomass.

## Experimental procedures

### Chemicals

Astaxanthin, 6‐BAP and all chemicals used for GC‐MS analyses were purchased from Sigma‐Aldrich (St. Louis, MO, USA). Trimethylamine was purchased from Fisher Scientific (Leicestershire, UK). The remaining chemicals were all obtained from Sinopharm Chemical Reagent Co., Ltd. (Shanghai, China) and were at least of analytical grade.

### Yeast strain and growth conditions

The astaxanthin‐overproducing mutant strain *X. dendrorhous* UV3‐721 was obtained from the wild‐type *X. dendrorhous* DSM 5626 (=*P. rhodozyma* ATCC 24202 and CBS 5905). DSM 5626 was first grown with nitrosoguanidine to obtain the mutant N1806‐04 (CGMCC No.3045). *X. dendrorhous* UV3‐721 was isolated from the medium after N1806‐04 was subjected to UV treatment. *X. dendrorhous* UV3‐721 was preserved in 20% glycerol at −80 °C and was maintained in YPD medium consisting of 20 g l^−1^ glucose, 10 g l^−1^ yeast extract and 20 g l^−1^ peptone. A single colony was inoculated into 250‐mL Erlenmeyer flasks containing 30 ml YPD medium and grown at 22 °C at a speed of 200 rpm for 48 h for seed culture. Subsequently, a 3‐ml seed culture was transferred into a 250‐ml Erlenmeyer flask containing 30 ml of fermentation medium and cultivated for 120 h according to the reported protocols (Pan *et al.*, 2017). Different concentrations of 6‐BAP (0.00, 0.10, 0.25, 0.50, 0.75, 1.00 mg l^−1^) were added to the fermentation medium at 24 h of cultivation. Cultures without 6‐BAP addition were used as controls for all other 6‐BAP‐added cultures. All cultures were sampled every 24 h for biomass, glucose concentration, ammonium concentration and metabolite analysis. For RNA preparation, all cultures were sampled at 36, 60 and 84 h, frozen by liquid nitrogen and stored at −80 °C before use. Each experiment was carried out in triplicate. The data were presented as the mean ± standards deviation of the three replicate cultures.

### Determination of biomass, glucose and ammonium concentration

Biomass was measured by dry cell weight (DCW, g l^−1^) according to the method by Pan *et al.* (2017). Glucose concentration in broth was analysed using an SBA‐40D biosensor (Shandong, China), and ammonium concentration was quantified with a LH‐N2 Reagent Kit (Lianhua Yongxing Science and Technology Co., Ltd, China) following the manufacturer's instructions.

### Astaxanthin extraction and quantification

Cell pellets were collected by centrifugation at 8000 *g* for 10 min at 4 °C. Astaxanthin was extracted from cell pellets with dimethylsulfoxide (DMSO) according to previous methods (Pan *et al.*, 2017).

Astaxanthin content was determined according to Xie *et al.* (2013) with moderate modifications. Briefly, astaxanthin was analysed on an Agilent 1200 series HPLC system equipped with a UV detector (Agilent Technologies, USA) and a YMC30 RP‐30 column (4.6 mm × 250 mm × 5 μm). The analysis procedure was performed at 25 °C with a 1 ml min^−1^ of mobile phase. The eluents were the following: (i) 3% double distilled water (ddH_2_O) in methanol containing 0.05 M ammonium acetate, and (ii) 100% tert‐butyl methyl ether. All eluents contained 0.1% (w v^−1^) butylated hydroxytoluene and 0.05% triethylamine. Elutions were carried out according to the following programme: isocratic at 3% B for 12 min followed by a linear gradient from 3% to 38% B in 1 min, isocratic at 38% B for 2 min, a linear increase to 68% B in 1 min and isocratic at 68% for 5 min followed by a linear decrease to 3% B in 4 min. The detection wavelength for the UV detector was adjusted to 478 nm.

### Preparation of intracellular metabolite samples and derivatization

The metabolome was prepared according to the procedures of Li *et al.* (2018) with moderate modifications. Briefly, 40 ml of each harvested sample was immediately mixed with 60 ml of prechilled methanol (−40 °C) to quench cells. The inactivated cells were collected by centrifugation at 8000 *g* for 10 min at 4 °C. The cell pellets were washed twice with physiological saline (0.9% of sodium chloride solution, prechilled at 4 °C) and stored at −80 °C before use. Next, 0.5 g of cells was homogenized in liquid nitrogen and extracted with prechilled methanol twice. The mixture was centrifuged at 8000 *g* for 10 min at −4 °C, and the supernatant was collected. Then, 50 μl of internal standard (adonitol in water, 0.2 mg ml^−1^) was mixed with the supernatants and the mixture was dried using a vacuum freeze‐dryer. Sample derivatization was performed according to the two‐stage technique (Yu *et al.*, 2016) with moderate modifications. Briefly, 60 μl of methoxyamine hydrochloride in pyridine (20 mg ml^−1^) was added to the dried sample and incubated at 37 °C for 2 h. Subsequently, the sample was silylated for 2 h at 37 °C by adding 60 μl of N‐methyl‐N‐trimethylsilyl‐trifluoroacetamide (MSTFA).

### GC‐MS‐based metabolomic analysis

Derivatized samples were analysed using a GC‐MS (Agilent Technologies 7890‐5975, Sacramento, USA) equipped with an HP‐5 MS capillary column (30 m × 250 μm × 0.25 μm, Agilent, Folsom, USA). Helium (constant flow: 1 ml min^−1^) was used as the carrier gas. One microlitre of derivatized sample was injected into the HP‐5 MS capillary column coated with 5% phenyl and 95% methylpolysiloxane in split mode with a split ratio of 1. The temperature of the injection port was set at 280 °C. The mass spectrometer was operated with the ion source and interface temperatures set at 200 °C and 280 °C respectively. The mass scan range was 50–600 m z^−1^. The oven temperature was set initially at 70 °C for 2 min and increased to 180 °C at a rate of 7 °C min^−1^. Then, it was continued to increase from 180 °C to 250 °C at the rate of 5 °C min^−1^, followed by a rate of 25 °C min^−1^ from 250 °C to 300 °C and was held at 300 °C for 8 min.

Peaks with high match values (> 750) and high levels of similarity (> 65%) were identified as metabolites. The relative levels of selected metabolites were determined through the characteristic ions of selected peaks. Metabolites were identified through comparisons with purified standards (Sigma). All detected peaks were identified by alignments of mass spectra to spectra in the library of the National Institute of Standards and Technology (NIST, Gaithersburg, MD, USA), and the relative abundance of identified metabolites was normalized by an internal standard and the biomass of the cells. As is commonly used for classification and biomarker selection in metabolomics studies, partial least squares‐discriminant analysis (PLS‐DA) was adopted to investigate metabolite profiling using SIMCA‐P software (version 11.0, Umetrics, Sweden) for multivariate statistical analysis, and hierarchical cluster analysis (HCA) was performed to analyse time‐course changes in the metabolites using Cluster software (HemI 1.0, Chinese Academy of Sciences, China).

### RNA isolation and relative quantification of transcripts

Samples for RNA extraction were collected in the experiment with and without the 6‐BAP treatment. Cells were quickly harvested after culturing for 36, 60 and 84 h, which were earlier than the sampling time for metabolite analysis, because the transcriptional responses were earlier than metabolic responses based on genetic central dogma. The Takara MiniBEST Universal RNA Extraction Kit (Cat# 9767, Lot# AK1401) was used to extract total RNA from yeast cells. First‐strand complementary DNA (cDNA) synthesis was performed by a High‐Capacity cDNA Reverse Transcription Kit (Applied Biosystems, USA) according to the manufacturer’s instructions. The quality and quantity of the obtained cDNA were measured using a Spectrophotometer (Quawell Nanodrop Q6000, USA) before the cDNA was diluted to 100 ng ml^−1^ for further RT‐qPCR analysis. The process of RT‐qPCR was performed with an Applied Biosystems real‐time PCR System. The expression level of each gene was normalized to that of the actin gene using the 2^−ΔΔCT^ method (Livak and Schmittgen, 2001). Primers for RT‐qPCR in this study were designed by Vector NTI^®^ Express Designer and are presented in Table S1.

### Analysis of intracellular reactive oxygen species (ROS) abundance

In this study, we used the one‐step Fluorometric Intracellular ROS Kit (Sigma‐Aldrich, MAK143‐KT) to detect intracellular ROS. The specific steps are as follows: yeast cells were sampled at different time points including 48, 72, 96 and 120 h. The cells were washed twice with 1 ml of phosphate‐buffered saline (PBS pH = 7.0) and resuspended in 100 μl of PBS. ROS abundance was measured by adding 100 μl of Master Reaction Mix (premixed with ROS detection reagent, DMSO and assay buffer) on a black 96‐well plate and incubating the cells in a 37 °C incubator for 1 h. Subsequently, ROS reacted with the fluorogenic sensor localized to the cytoplasm, resulting in a fluorometric product (λex = 490/λem = 520 nm) proportional to the amount of ROS present. Fluorescence was read using a fluorescence microplate reader (Molecular Devices Gemini EM, USA). For each time point, at least 100 cells were examined.

### Statistical analysis

In this study, all the tests were carried out in three biological replicates and two technical repeats were set for GC‐MS and RT‐qPCR measurements. Data in the figures or tables were shown as the averages of 6 replicates, and error bars in the figures represent standard errors. For all data analysis, two‐tailed *t*‐tests were performed, and *P* values < 0.05 were considered to be statistically significant.

## Conflict of interest

None declared.

## Author contributions

XP, XL and YL conceived and designed the experiments; XP performed the experiments and wrote the manuscript; XP, RD and JL analysed the data; BW and JJ revised the manuscript; WX and CC performed ROS analysis; XH, GZ modified the study and assisted in performing the experiments. XL and YL contributed reagents/materials/analysis tools. All authors read and approved the manuscript.

## Supporting information


**Fig. S1.** Effect of 6‐BAP on biomass and astaxanthin content of *Xanthophyllomyces dendrorhous* UV3‐721. (A) 6‐BAP fed at the beginning of cultivation, (B) 6‐BAP fed at 24 h of cultivation. The maximum astaxanthin content was achieved, when adding 0.25 mg L^‐1^ 6‐BAP at 24 h. No 6‐BAP was added into the control. Values are mean ± standard deviation of three independent experiments.
**Fig. S2.** The mechanisms through with 6‐BAP addition effects the cells of *Xanthophyllomyces dendrorhous*.

**Table S1.** Gene‐specific primers used for RT‐qPCR.
