# Supplementary material for: Enhancing astaxanthin accumulation in Xanthophyllomyces dendrorhous by a phytohormone: metabolomic and gene expression profiles
Source: Microb Biotechnol. 2020 May 19;13(5):1446–60. doi: 10.1111/1751-7915.13567 (PMC7415379; doi:10.1111/1751-7915.13567)
Supplement: Supplementary file 1 — Fig. S1. Effect of 6‐BAP on biomass and astaxanthin content of Xanthophyllomyces dendrorhous UV3‐721. (A) 6‐BAP fed at the beginning of cultivation, (B) 6‐BAP fed at 24 h of cultivation. The maximum astaxanthin content was achieved, when adding 0.25 mg L‐1 6‐BAP at 24 h. No 6‐BAP was added into the control. Values are mean ± standard deviation of three independent experiments. Fig. S2. The mechanisms through with 6‐BAP addition effects the cells of Xanthophyllomyces dendrorhous. [file MBT2-13-1446-s001.docx]

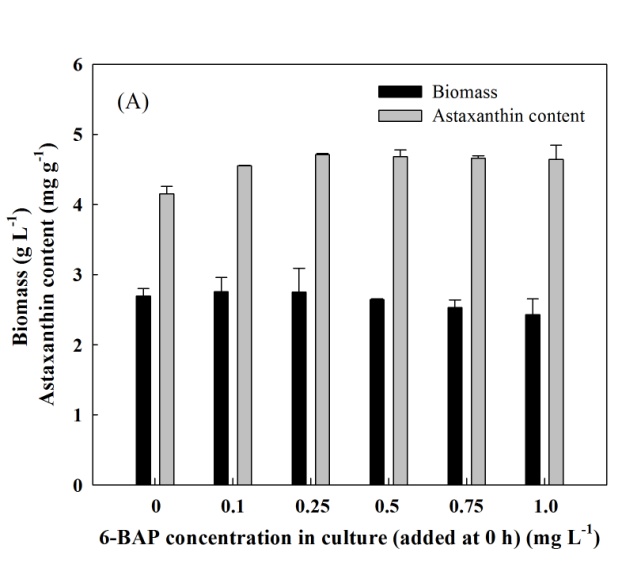

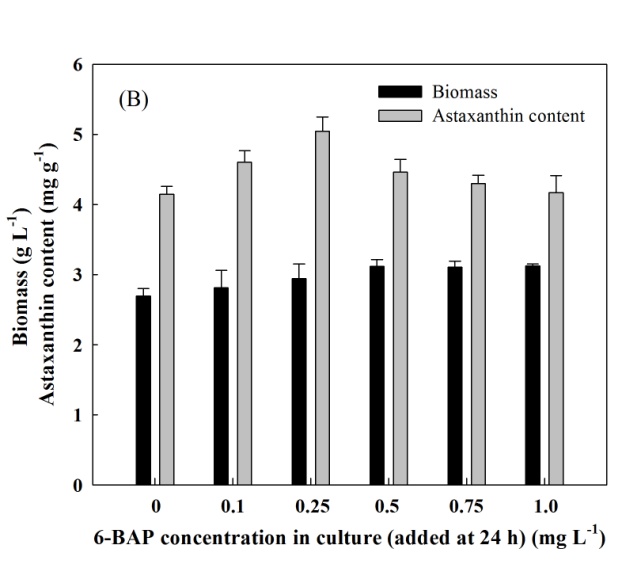


**Fig. S1** Effect of 6-BAP on biomass and astaxanthin content of *X. dendrorhous* UV3-721. (A) 6-BAP fed at the beginning of cultivation; (B) 6-BAP fed at 24 h of cultivation. The maximum astaxanthin content was achieved, when adding 0.25 mg L^-1^ 6-BAP at 24 h. No 6-BAP was added into the control. Values are mean ± standard deviation of three independent experiments.

**Fig. S2** The mechanisms through with 6-BAP addition effects the cells of *X. dendrorhous*. PYR: Pyruvate; OA: Oxaloacetate; IC: Isocitrate; OG: 2-oxo-glutarate; Asta: Astaxanthin; FA: Fatty acids.
