# Supplementary material for: Enhancing astaxanthin accumulation in Xanthophyllomyces dendrorhous by a phytohormone: metabolomic and gene expression profiles
Source: Microb Biotechnol. 2020 May 19;13(5):1446–60. doi: 10.1111/1751-7915.13567 (PMC7415379; doi:10.1111/1751-7915.13567)
Supplement: Supplementary file 2 — Table S1. Gene‐specific primers used for RT‐qPCR. [file MBT2-13-1446-s002.docx]

| Table S1 Gene-specific primers used for RT-qPCR; F: Forward; R: Reverse. | |  |
| --- | --- | --- |
| Gene Name | Primers: F | Primers: R |
| *hmgR* (HMG-CoA reductase) | 5’-GCTCTCCTCTGCTCATCCAATCGA-3’ | 5’-TGGATCCCAAAGAGAAGAACGCATC-3’ |
| *idi* (IPP isomerase) | 5’-ATGTCCATGCCCAACATTGTTCCC-3’ | 5’-TGATGTTGGACATCAAGTGGCAGGT-3’ |
| *crtE* (GGPP synthase) | 5’-GATTACGCGAACATCCTCACAGCAA-3’ | 5’-CAACAACGTTCTGGATGACCTCGAG-3’ |
| *crtYB* (Phytoene synthase) | 5’-ACGGCTCTCGCATATTACCAGATCC-3’ | 5’-CGCCATTTCTGATGATCCATGAGTC-3’ |
| *crtI* (Phytoene dehydrogenase) | 5’-CACAGCTATCATCGTGGGATGTGG-3’ | 5’-ATCTGGCAAGAGCAGCAAACTGGG-3’ |
| *crtS* (Astaxanthin synthase) | 5’-TCATCTTGGTCTTGCTCACAGGTGC-3’ | 5’-TCGCATGCTCTTCACCTGTACGAG-3’ |
| *act* (Actin) | 5’-CCGCCCTCGTGATTGATAAC-3’ | 5’-TCACCAACGTAGGAGTCCTT-3’ |
